# Liver resection versus transarterial chemoembolization for the treatment of intermediate‐stage hepatocellular carcinoma

**DOI:** 10.1002/cam4.2038

**Published:** 2019-03-12

**Authors:** Shuling Chen, Huilin Jin, Zihao Dai, Mengchao Wei, Han Xiao, Tianhong Su, Bin Li, Xin Liu, Yu Wang, Jiaping Li, Shunli Shen, Qi Zhou, Baogang Peng, Zhenwei Peng, Sui Peng

**Affiliations:** ^1^ Division of Interventional Ultrasound The First Affiliated Hospital of Sun Yat–sen University Guangzhou China; ^2^ Department of Liver Surgery The First Affiliated Hospital of Sun Yat–sen University Guangzhou China; ^3^ Department of Gastroenterology and Hepatology The First Affiliated Hospital of Sun Yat–sen University Guangzhou China; ^4^ Clinical Trials Unit The First Affiliated Hospital of Sun Yat‐sen University Guangzhou China; ^5^ Department of Interventional Oncology The First Affiliated Hospital of Sun Yat‐sen University Guangzhou China; ^6^ Department of Oncology The First Affiliated Hospital of Sun Yat–sen University Guangzhou China

**Keywords:** intermediate‐stage hepatocellular carcinoma, liver resection, Markov Model, propensity score matching, transarterial chemoembolization

## Abstract

**Background:**

The role of transarterial chemoembolization (TACE) as the standard treatment for intermediate‐stage hepatocellular carcinoma (HCC) is being challenged by increasing studies supporting liver resection (LR); but evidence of survival benefits of LR is lacking. We aimed to compare the overall survival (OS) of LR with that of TACE for the treatment of intermediate‐stage HCC in cirrhotic patients.

**Methods:**

A Markov model, comparing LR with TACE over 15 years, was developed based on the data from 31 literatures. Additionally, external validation of the model was performed using a data set (n = 1735; LR: 701; TACE: 1034) from a tertiary center with propensity score matching method. We conducted one‐way and two‐way sensitivity analyses, in addition to a Monte Carlo analysis with 10 000 patients allocated into each arm.

**Results:**

The mean expected survival times and survival rates at 5 years were 77.8 months and 47.1% in LR group, and 48.6 months and 25.7% in TACE group, respectively. Sensitivity analyses found that initial LR was the most favorable treatment. The 95% CI for the difference in OS was 2.42‐2.46 years between the two groups (*P* < 0.001). In the validation set, the 5‐year survival rates after LR were significantly better than those after TACE before (40.2% vs. 25.9%, *P* < 0.001) and after matching (43.2% vs 30.9%, *P* < 0.001), which was comparable to the model results.

**Conclusions:**

For cirrhotic patients with resectable intermediate‐stage HCC, LR may provide survival benefit over TACE, but large‐scale studies are required to further stratify patients at this stage for different optimal treatments.

## 1 INTRODUCTION

Intermediate‐stage hepatocellular carcinoma (HCC) can be moderately controlled with appropriate treatments.^1^, ^2^ However, the management strategies for intermediate‐stage HCC remain controversial without a global consensus.^3^, ^4^, ^5^, ^6^, ^7^, ^8^ The Barcelona Clinic Liver Cancer staging system, endorsed by many HCC associations, recommends liver resection (LR) for very‐early and early‐stage HCC, while recommending transarterial chemoembolization (TACE) for intermediate‐stage HCC.^3^, ^6^, ^9^, ^10^ It seems that patients with intermediate‐stage tumors are not the candidates for curative treatment. On the other hand, the role of TACE was established by one randomized controlled trial (RCT), as well as a meta‐analysis with the demonstration of improved survival of TACE compared to the best available supportive care. However, these did not compare TACE to other treatment modalities such as resection.^11^, ^12^ Moreover, observational studies from Eastern and Western countries have emerged to show that LR was safer and yielded better survival than TACE for selected patients with intermediate‐stage HCC.^4^, ^5^, ^7^, ^8^ Simultaneously, several guidelines and consensuses have come to state that tumor multifocality is not a contraindication to LR Some Asian studies even recommend LR for intermediated‐stage HCC.^13^, ^14^, ^15^, ^16^, ^17^ However, these observational studies were conducted with non‐negligible biases, and the guidelines/consensuses were largely based on expert opinions. To date, only a very few studies have been performed to directly compare LR with TACE for at this stage.^18^, ^19^, ^20^, ^21^ Among them, none have been designed well enough to draw robust conclusion until an RCT was reported. Yin et al revealed the significant superiority of LR over TACE in a population of 173 patients.^22^ Despite this, the study was far from offering a definitive conclusion due to its small sample size and attribution of a single center study. New multicenter RCTs with a large sample size are required urgently in this respect. However, such large trials are difficult to conduct due to the difficulties of patient enrollment and treatment allocations. The Markov model is capable of estimating a disease's outcome by simulating disease progression where patients move through different health states over the preset cycles. It has been successfully applied to the simulation of a head‐to‐head comparison of treatment efficacy with large sample sizes for a long follow‐up period.^23^, ^24^ Cho YK et al have compared LR with RFA in the treatment of very‐early stage HCC by constructing a Markov model with hypothetical cohorts of 30,000 patients for a 15‐year follow‐up period.^23^ Thus, we aimed to simulate a trial that compares LR with TACE in terms of overall survival (OS) for cirrhotic adult patients with intermediate‐stage HCC by constructing a Markov model, and then externally validate the model with a large data set using a propensity score matching (PSM) method.

## 2 METHODS

### 2.1 Model Construction

**Figure 1:**
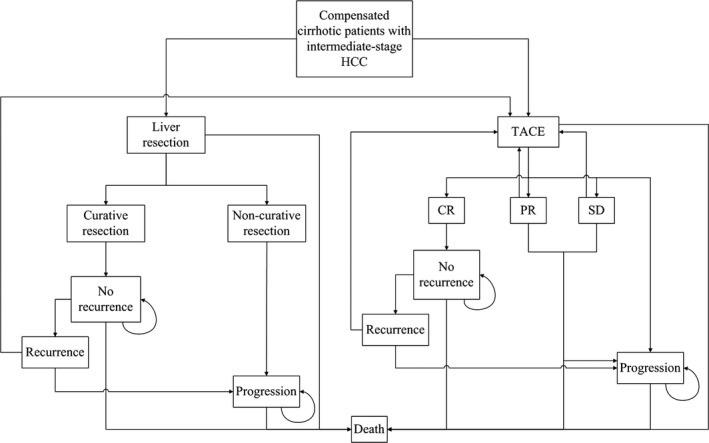
Flow diagram of the Markov cohort model. Each pane represents a state of health. Straight lines with arrows indicate transition from one state to another one while circular arrows mean that some patients may stay at the same state for more than one cycle. Two therapy strategies were designed in this model with initial treatments of LR and TACE. Patients with progressive HCC in both groups were assumed to receive no further treatments. For TACE group, patients with PR, SD, or recurrence after CR will be considered candidates for repeated TACE except those with progressive disease. For LR group, patients with recurrent HCC after initial LR were assumed to receive repeated TACE treatment. Patients with positive resection margin will be assumed to have progressive HCC. LR, liver resection; TACE, transarterial chemoembolization; HCC, hepatocellular carcinoma; PR, partial response; SD, stable disease; CR, complete response

To simulate a RCT with a follow‐up period of 15 years, a Markov model with multistate was constructed to compare the treatment efficacy of LR and TACE for intermediate‐stage HCC. In this model, two therapeutic decisions were deployed. Death was designated as the terminal state and OS was selected as the study end point. Details of the model were shown in Figure 1.

As shown in the model, 15 Markov states in total were developed, including seven states in the LR group, and the remaining eight in the TACE group. The cycle length was set to be 1 year, which was in accord with the pertinent interval for observing the treatment response of tumor episodes. Furthermore, to obtain sustained outcomes and the actual life expectancy of cirrhotic patients, we assumed a 15‐years’ observation period for the patients.^25^ The half‐cycle correction was adopted in this study.^26^ The annual mortality and recurrence rates were derived from the median survival or cumulative probability of survival, through the declining exponential approximation of life expectancy (DEALE) method.^27^ The TreeAge‐Pro‐2008 software (TreeAge Software Inc, Williamstown, MA, USA) was employed for Markov model construction.

To evaluate the contribution of variables to the both the OS and the robustness of our results, sensitivity analyses were also performed.^28^ One‐way and two‐way sensitivity analyses were first performed followed by the second‐order Monte Carlo probabilistic sensitivity analysis to estimate the potential impact of parameter uncertainties on the model results, with 10,000 patients allocated into each group respectively.

### 2.2 Literature selection

All the transition probabilities applied in this model were retrieved from the included English publications that explored the treatment efficacy of LR^7^, ^19^, ^21^, ^22^, ^29^, ^30^, ^31^, ^32^, ^33^, ^34^, ^35^, ^36^, ^37^ and/or TACE^12^, ^38^, ^39^ for intermediate‐stage patients, in addition some other common estimations in this model (Table S1, S2, S3).^47^, ^48^ PubMed and Cochrane Library were selected for literature retrieval with the latest searching on July 01, 2016. Search terms were as follow: hepatocellular carcinoma, liver cancer, primary liver carcinoma, liver cell carcinoma, hepatectomy, liver resection, liver surgery, hepatic resection, resection, surgery, surgical therapy, transarterial chemoembolization, TACE, transarterial embolization, TAE. Reference lists from the selected studies were hand‐searched to further identify relevant trials. More details are provided in Supplementary Methods.

### 2.3 Parameter estimation

To guarantee variance stabilization, the extracted transition probabilities were subjected to double arcsine transformations before pooling.^55^ The Wilson score method was also used to estimate the 95% confidence intervals (CIs) of these probabilities.^56^ STATA software (Stata Corp., College Station, TX, USA) was then employed to integrate the above rates through a random‐effect model. Besides, SAS 9.2 (SAS Institute Inc, Cary, NC) was adopted to apply the Wilson score method and calculate the 95% CIs. Corresponding details for this part were presented in Supplementary Methods.

### 2.4 Summary of transition probabilities and assumptions

The transition probabilities extracted from the literatures were summarized in Table 1. For disease‐free patients, the estimated annual mortality was derived by summing up the annual mortality of general population and the liver‐related annual mortality of cirrhotic patients.^23^ The average age of patients in the selected studies ranged from 46.0 years to 72.5 years, then the mean age of this cohort was assumed to be 60‐65 years with the annual age‐related mortality of 0.055.^48^ With regard to the non‐liver related and tumor‐free liver related annual mortality, they were estimated as what Cho YK et al have reported.^23^ More details are illustrated in Supplementary Methods.

**Table 1:** Estimated transition probabilities extracted from literatures for the Markov Model

Variable	LR	TACE
Annual mortality rate of general population(60‐65 years old)	0.055^48^	
Annual mortality rate of cirrhotic patients^a^	0.027^47^	
Annual mortality rate for progressive HCC^a^	0.746 (0.573‐0.843)^49^, ^50^	
Probability of preoperative mortality	0.033 (0.011‐0.054)^7^, ^19^, ^21^, ^22^, ^31^, ^36^	—
Probability of incomplete resection	0.114 (0.06‐0.216)^22^, ^32^, ^34^	—
Probability of annual recurrence^b^	0.245 (0.186‐0.303)^29^, ^30^, ^34^, ^35^	—
Probability of progression of recurrent HCC	0.235 (0.224‐0.243)^33^, ^36^	—
Probability of CR after TACE	—	0.156 (0.025‐0.406)^12^, ^38^, ^39^, ^40^
Probability of PR after TACE		0.494 (0.268‐0.725)^12^, ^38^, ^39^, ^40^
Probability of SD after TACE	—	0.202 (0.072‐0.263)^12^, ^38^, ^39^, ^40^
Probability of PD after TACE	—	0.148 (0.090‐0.269)^12^, ^38^, ^39^, ^40^
Probability of recurrence of CR patients within 1 year	—	0.663 (0.576‐0.750)^44^, ^46^
Probability of CR transforming into PD within 1 year	—	0.127^44^, ^46^
Probability of PR transforming into PD within 1 year	—	0.127^44^, ^46^
Probability of SD transforming into PD within 1 year	—	0.127^44^, ^46^

LR, liver resection; TACE, transarterial chemoembolization; HCC, hepatocellular carcinoma; CR, complete response; SD, stable disease; PD, progressive disease; PR, partial response.

a Probabilities were converted into annual mortality applying the declining exponential approximation of life‐expectancy approach: *μ* = 1‐ (r),^1^ ^/time^ where r referred to survival rate or 50% and time referred to observation time or median survival time (years).

b The annual recurrence probability was derived from post LR 5‐year cumulative recurrence rate by the assumption of DEALE formula as described above; 5‐year disease‐free survival was also transformed into annual recurrence rates applying the declining exponential approximation of life‐expectancy method: *μ* = −1/t*ln(s), where t referred to the follow‐up time and s referred to data extracted from literatures.

### 2.5 External validation

Finally, the external validation of this model was performed by comparing it with the survival data from a retrospective study on 1735 consecutive intermediate‐stage patients with resectable HCC who received LR (n = 701) or TACE (n = 1034) as an initial therapy in the First Affiliated Hospital of Sun Yat‐sen University from January 2008 to February 2014. To reduce the potential bias in this study, a PSM method was performed to make adjustments for the differences in baseline characteristics of two arms. All the baseline variables listed in Table 2 were entered in the full nonparsimonious model to perform matching. The follow‐up was censored on December 2015. LR and TACE were performed respectively according to the protocols we reported previously.^57^, ^58^ The follow‐up protocol was conducted as we previously described.^58^ The detailed study design is described in Supplementary Methods.

**Table 2:** Baseline characteristics of patients

Variable	Before matching			After matching		
	LR (n = 701)	TACE (n = 1034)	*P* value	LR (n = 623)	TACE (n = 623)	*P* value
Age, y	51 (22‐75)	54 (23‐75)	0.100	51 (23‐75)	51 (23‐75)	0.898
Sex(M/F)	652/49	963/71	0.921	590/33	588/35	0.901
Hepatitis B (±)	645/56	950/84	0.919	601/22	601/22	0.999
Hepatitis C (±)	21/680	26/1008	0.545	16/607	13/610	0.708
Cirrhosis(yes/no)	617/84	889/145	0.218	599/24	595/28	0.671
Child Pugh classification (A/B)	689/12	1001/33	0.057	615/8	615/8	0.999
ECOG status (0/1)	658/43	960/74	0.436	601/22	598/25	0.767
Portal hypertension (yes/no)	182/519	291/743	0.323	165/458	156/467	0.604
R15, %			0.068			0.810
≤10	645	924		585	588	
>10	56	110		38	35	
Tumor size, cm			0.168			0.818
≤5	266	358		265	260	
>5	435	676		358	363	
Tumor number			0.127			0.999
2	331	449		330	330	
>2	370	585		293	293	
AFP, ng/mL			0.159			0.894
≤400	170	221		150	147	
>400	531	813		473	476	
GGT, u/L	430.2 (33.5‐690.1)	456.2 (25.1‐622.7)	0.512	430.0 (33.5‐622.7)	431.0 (33.1‐622.7)	0.870
ALT, u/L	25.5 (13.0‐66.0)	28.2 (11.0‐74.8)	0.154	26.5 (13.0‐66.0)	26.5 (13.0‐65.3)	0.914
Albumin, g/L	36.3 (34.8‐42.5)	35.9 (34.0‐47.3)	0.251	35.5 (34.9‐42.5)	35.5 (34.8‐42.5)	0.900
TBIL, umol/L	11.4 (6.3‐28.5)	12.5 (5.5‐34.2)	0.311	11.6 (6.5‐28.5)	11.7 (6.7‐28.5)	0.943
Creatinine, umol/L	67.5 (38.7‐99.5)	70.3 (47.8‐95.0)	0.685	68.9 (48.8‐94.5)	68.9 (48.8‐95.0)	0.889
Platelet count, 109/L	121.2 (92.0‐320.0)	118.5 (75‐345)	0.096	119.0 (92.0‐320.0)	119.0 (92.0‐320.0)	0.980
Hemoglobin, g/L	126.0 (112.0‐150.0)	124.0 (105.0‐153.0)	0.647	125.0 (113.0‐149.0)	125.0 (114.0‐149.0)	0.923
White blood cell, 109/L	5.5 (4.5‐8.0)	5.8 (4.3‐9.0)	0.185	5.6 (4.6‐8.0)	5.6 (4.6‐8.0)	0.899
Distribution of tumor			0.977			0.999
Right liver	412	607		405	405	
Left liver	41	62		36	36	
Both liver	248	365		182	182	
Follow‐up time (median, months)	32.0 (1‐96)	31.0 (1‐96)	0.982	32.0 (1‐96)	32.0 (1‐96)	0.996

There was no significant difference in average age between LR (51 years) and TACE (54 years) groups. Hepatitis B virus infection was the major etiology of HCC while Hepatitis C virus was the minor one. Most of the patients have developed cirrhosis and cirrhotic patients were mainly in the classification of Child‐pugh A.

LR, liver resection; TACE, transarterial chemoembolization; GGT, γ‐glutamyltranspeptidase; ALT, alanine amino transferase; TBIL, total bilirubin; ICGR15, indocyanine green retention rate in 15 minutes; AFP, alpha‐fetoprotein; ECOG, Eastern Cooperative Oncology Group.

## 3 RESULTS

### 3.1 Survival outcomes of the model

**Figure 2:**
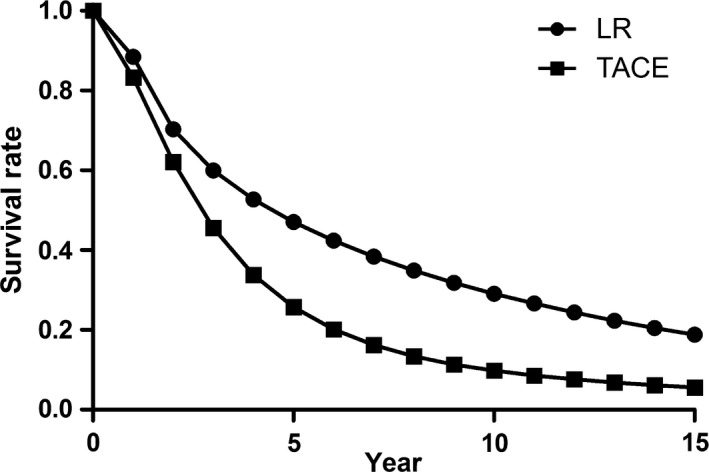
Overall survival curves for LR and TACE groups in the treatment of compensated cirrhotic patients with intermediate‐stage HCC. The survival curves of the LR group were better than that of the TACE group. LR, liver resection; TACE, transarterial chemoembolization; HCC, hepatocellular carcinoma

From the Markov model, the expected OS was 6.49 years (77.8 months) and 4.05 years (48.6 months) for LR and TACE groups, respectively (Figure 2). The estimated survival rates at 1, 3, and 5 years were 88.5%, 60.0%, and 47.1% in the LR group, and 83.3%, 45.4%, and 25.7% in the TACE group.

### 3.2 One‐way and two‐way Sensitivity analyses

One‐way sensitivity analyses for all the parameters showed that the curves of expected survival for LR treatment were always above those for TACE without crossing points (Figure S1). This suggested that initial LR always had the survival benefit over TACE. Similar results could be observed in two‐way sensitivity analyses (Table S4). For any of the two variables, there was no point of intersection between these two therapies as shown in the figures, even when comparing the most sensitive factors in both groups and assuming the best case scenario for TACE. Tornado diagrams showed that the top three sensitive factors for survival in LR group were disease‐free survival, perioperative mortality, and incomplete resection rate, while the top three factors in the TACE group were CR rate, progressive disease (PD) rate, and probability of partial response (PR) transited to PD (Figure S2). All the above factors were associated with the corresponding initial treatment. Thus, the results of Tornado diagram were consistent with those of the sensitivity analyses which showed the survival outcomes were more sensitive to variables related to initial treatment options. Detailed results of sensitivity analysis were described in Supplementary Results.

### 3.3 Second‐order Monte Carlo simulation

Using Monte Carlo simulation, the probability distributions of OS demonstrated that the estimated OS in the LR group (95% CI: 6.47‐6.51 years) was better than that in the TACE group (95% CI: 4.04‐4.06 years) (Figure S3). The 95% CIs of the difference in OS between these two groups were 2.42‐2.46 years with statistically significant difference (*P* < 0.001). This finding further verified our model by showing that the superiority of LR to TACE would not be changed by the uncertainties of parametric estimations.

### 3.4 Results from the overall validation cohort

There were no significant differences in baseline characteristics between LR and TACE groups (Table 2). Other detailed perioperative and operative data were showed in Supplementary Results, Table S5 and S6.

**Figure 3:**
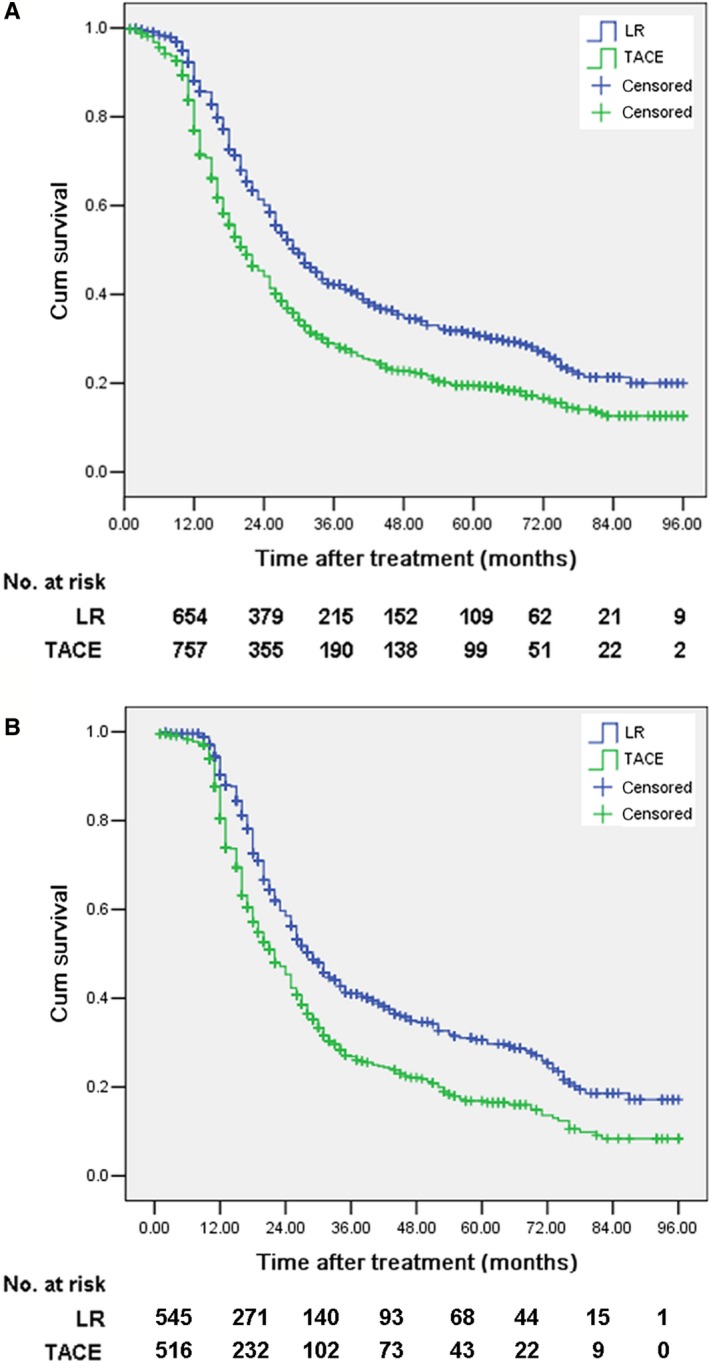
Cumulative survival curves of LR group and TACE group for the validation cohort patients with intermediate‐stage HCC before (A) and after (B) matching. Note the significant differences of cumulative survival rate, median survival time and survival proportion at 5‐year between LR group and TACE group. LR, liver resection; TACE, transarterial chemoembolization; HCC, hepatocellular carcinoma

For the overall cohort, the 1‐, 3‐, and 5‐year cumulative survival rates, median survival and 5‐year survival proportion in the LR group were 88.1%, 44.2%, 31.8%, 44.2 months and 40.2%, respectively (Figure 3A). Similarly, the corresponding data in the TACE group were 77.0%, 29.1%, 18.6%, and 33.7 months and 25.9%, respectively (Figure 3A). The differences of the above survival outcomes between these two groups were statistically significant (*P* < 0.001).

### 3.5 Results from the propensity score‐matched validation cohort

For the 623 propensity score‐matched pairs, there were no significant differences in baseline characteristics between LR group and TACE group (Table 2). Other detailed perioperative and operative data were showed in Supplementary Results, Table S5 and S6.

The 1‐, 3‐, and 5‐year cumulative survival rates, median survival, and 5‐year survival proportion after LR were 90.3%, 41.3%, 30.6%, 43.3 months, and 43.2% respectively, while the corresponding values for TACE were 80.5%, 26.9%, 16.9%, 32.6 months, and 30.9%, respectively (Figure 3B).

### 3.6 Multivariable analysis

Multivariable analysis presented that tumor number (hazard ratio (HR)=1.238, 95% CI: 1.095‐1.400, *P* = 0.002), alpha‐fetoprotein (AFP) (HR = 1.485, 95% CI:1.237‐1.783, *P* < 0.001), and the type of initial treatment (HR = 1.497, 95% CI: 1.328‐1.688, *P* < 0.001) was the significant factors for OS (Table S7). The median survival and survival proportion at 5 years after LR were significantly better than those after TACE in our center, which was consistent with the superior trend of LR over TACE as represented in our model. Moreover, the survival proportions at 5 years after both treatments in our clinical data (LR, 40.2%; TACE, 25.9%) seemed to be comparable to those simulated by our model (LR, 42.4%; TACE, 26.8%).

## 4 DISCUSSION

The role of TACE as the standard therapy for intermediate‐stage HCC is being challenged by increasing studies which show that LR is safe and feasible with better survival outcomes than TACE.^5^, ^29^, ^34^, ^59^, ^60^ However, controversy still surrounds the selection of LR or TACE due to the lack of convincing evidences from well‐conducted RCTs. The Markov model can be used to predict disease outcomes and compare treatment efficacy and cost‐effectiveness of different therapies.^23^, ^62^, ^63^ Based on the constructed model representing patients’ disease courses and transition probabilities, representing probabilities moving from one state to another, the OS of each arm was calculated by running the whole model for preset cycles. Influencing factors of OS can also be identified through sensitivity analysis. Therefore, it may help provide informative data on the comparison of LR and TACE for intermediate‐stage patients when RCTs are unavailable.

The present study demonstrates that LR can provide better OS for patients with intermediate‐stage HCC, compared to TACE based on the Markov model. Moreover, the model's validity was further confirmed by the balanced survival data from our center with the PSM method. This might be explained by the fact that LR is the curative therapy with potential to remove almost all the macroscopic lesions, whereas TACE is a down‐stage therapy, inevitably leaving viable tumor cells left in the liver parenchyma. If a patient in intermediate stage is technically resectable, a better survival than that of TACE can be theoretically expected. The only available RCT from China reported the median OS of 41 months and 14 months in LR and TACE groups respectively.^22^ It appeared that, for patients receiving LR, our model presented a slightly more satisfactory outcome than our center's results in addition to other previous literature. Explanations for this may be as follows. First, the Markov cycle was repeated 15 times which meant that our follow‐up period reached a length of 15 years that was long enough to obtain data from nearly every patient. Nevertheless, the follow‐up ranges in real clinical studies were hardly longer than 10 years. In this case, some patients with long‐life duration would have been lost and then the median OS would be lowered. For instance, the survival rate at 5 years in our center (43.2%) was very close to that in the model (47.1%); however the median OS in our center was relatively shorter (43.3 vs 77.8 months). Second, we have assumed the annual mortality rates of both general population and cirrhotic patients due to liver‐related disease to be constant. However, the mortality rate may increase as the time goes by and cirrhosis progresses. The current setting might more or less lengthen the survival for both groups in our model. Third, the patients’ survival in reality could be influenced by various clinical and socioeconomic factors whereas our model is a simplified simulation with the ideal assumption. Regarding the outcome after TACE, the median OS of 33.1 months for patients in our center was consistent with the results reported by previous studies (3.4 to 48 months),^20^, ^22^, ^64^, ^65^ and the model result of 48.6 months was generally much longer than those of the above studies. However, it seems reasonable to have such a model result because high OS (40 to 48 months) has already been reported by studies in Japan and Europe.^64^, ^67^, ^68^ There is wide variability in TACE protocols such as conventional TACE (cTACE) and drug‐eluting bead TACE. The literature regarding TACE in our model included all the studies investigating cTACE, and such a satisfactory model result might partly due to the best scenario given to TACE and long follow‐up period. TACE represents a heterogeneous treatment modality, which partly explains the varied survival durations after TACE.^69^ Similar to our study, the efficacy of cTACE in our center was relatively poorer than that in the model, represented by the lower CR and PR rates and higher SD and PD rates. On the whole, the model's results were consistent with the survival data in reality and the Markov model can extend the results of clinical trials with larger sample sizes and longer follow‐up periods.

There are several limitations to this study. First and foremost, we should note that the comparison of these two competing treatment modalities was feasible only when tumors were resectable and both treatments were applicable. However, in clinical practice, a certain proportion of intermediate‐stage patients may not be suitable to receive LR due to many high‐risk factors such as the presence of portal hypertension, an insufficient liver remnant or other comorbidities. Second, it is acknowledged that patients with intermediate‐stage HCC are heterogeneous regarding tumor burden and liver function, and the treatment might vary in different subtypes of this population.^70^ However, subgroup analysis according to the liver function and tumor burden was not performed, which could not provide further information on the stratification of these patients for more specific treatment allocation. This limitation is unfortunately unavoidable for the following two reasons. One is the absence of literature reporting the parameters we want in the subgroup analysis. The other is the impossibility to extract data respectively in different subgroups, due to the fact that some articles reported the results in a single group. Third, although we performed a detailed systematic review of all the eligible RCT and observational studies to obtain data, the paucity of RCTs included might influence the results to some extent. Fourth, some parameter estimations were based on a very limited number of studies. For instance, the recurrence rate after CR and the progressive rate of recurrent HCC were both obtained from only two studies. Moreover, our model's hypothetic cohort was cirrhotic patients, whereas the included articles in which we extracted transition probabilities enrolled a small part of patients with chronic hepatitis, but without cirrhosis. Due to the acceptable proportion of these patients, our findings have not been significantly influenced, which was also confirmed by the results of the second‐order Monte Carlo simulation and our own data set. Fifth, we estimated the OS from the Markov model by simulating the whole HCC disease course from the first treatment to death; however, we confined all the potential treatments to LR or TACE. In reality, many alternative treatments are available to treat residual lesions and recurrence. For example, subsequent comprehensive treatments with radiofrequency ablation, percutaneous ethanol injection, or sorafenib might collectively lead to a different prognosis. Nevertheless, it is unrealistic to expect a model simulating exactly the real clinical situations because too many uncertainties concerning the treatment selection throughout a patient's disease history are present. Our model does not aim to completely replace RCTs, but only tries to provide informative data in these provocative research hotspots.

Based on the Markov model with an enormous sample size and a long follow‐up period, and our own clinical data after balancing patient and tumor characteristics, our findings may provide critical information for the future investigations and management of intermediate‐stage HCC. LR is superior to TACE regarding the OS of compensated cirrhotic patients with resectable intermediate‐stage HCC. Future large‐scale high‐quality studies are required to stratify patients at this stage for different optimal treatments.

## CONFLICT OF INTEREST

All coauthors have seen and agreed with the content of the manuscript and there is no financial interest to report.
