# Supplementary figures and images for: Liver resection versus transarterial chemoembolization for the treatment of intermediate‐stage hepatocellular carcinoma
Source: Cancer Med. 2019 Mar 12;8(4):1530–9. doi: 10.1002/cam4.2038 (PMC6488138; doi:10.1002/cam4.2038)

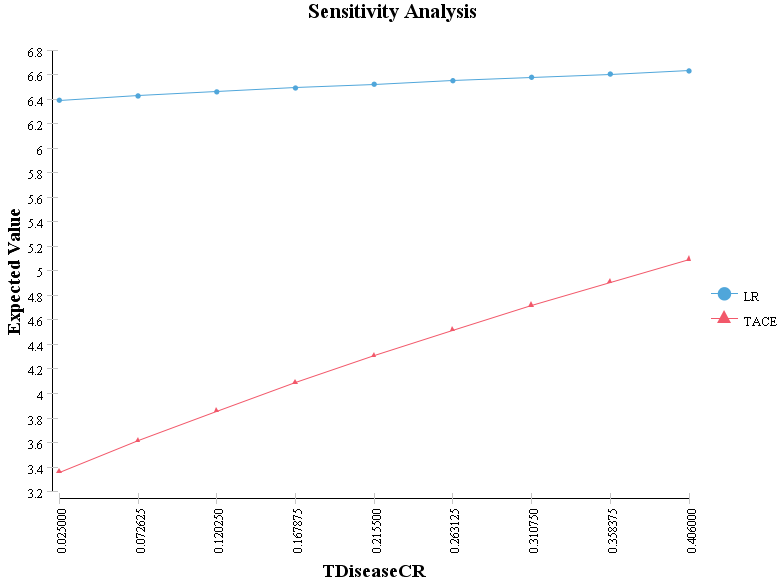

Supplement: Supplementary file 1 [file CAM4-8-1530-s001.tif]

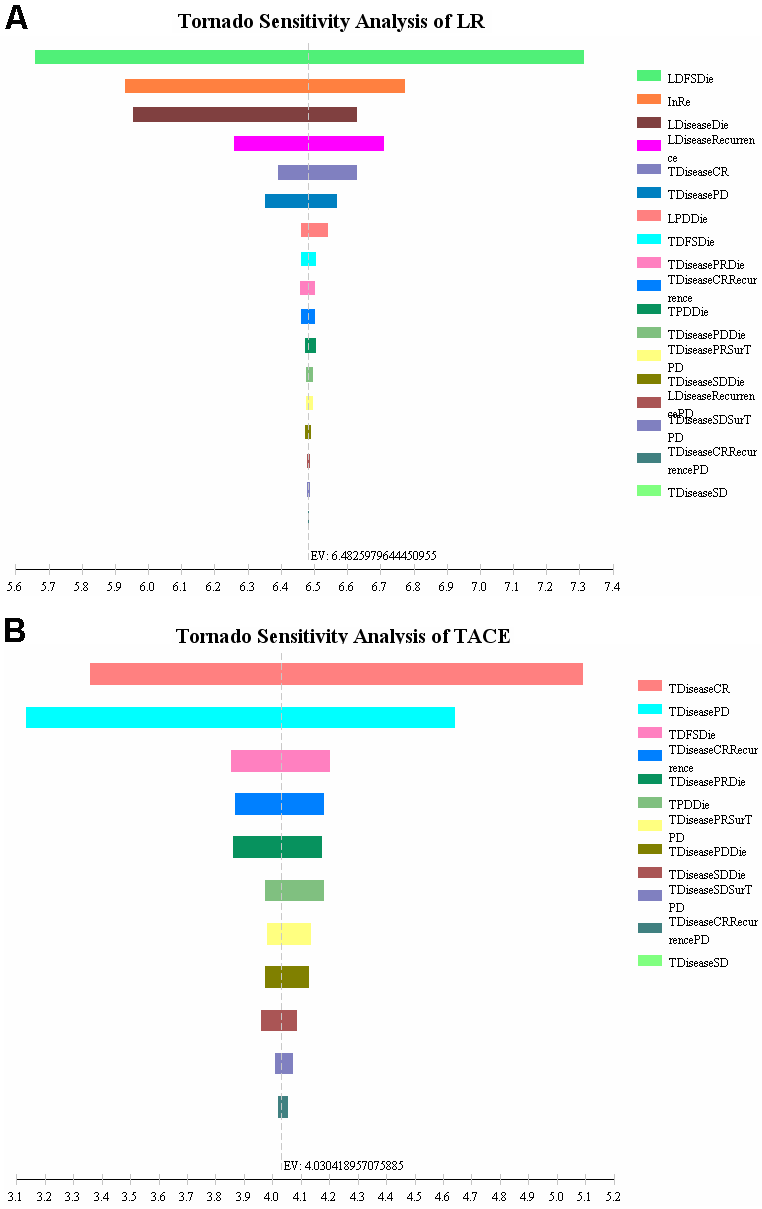

Supplement: Supplementary file 2 [file CAM4-8-1530-s002.tif]

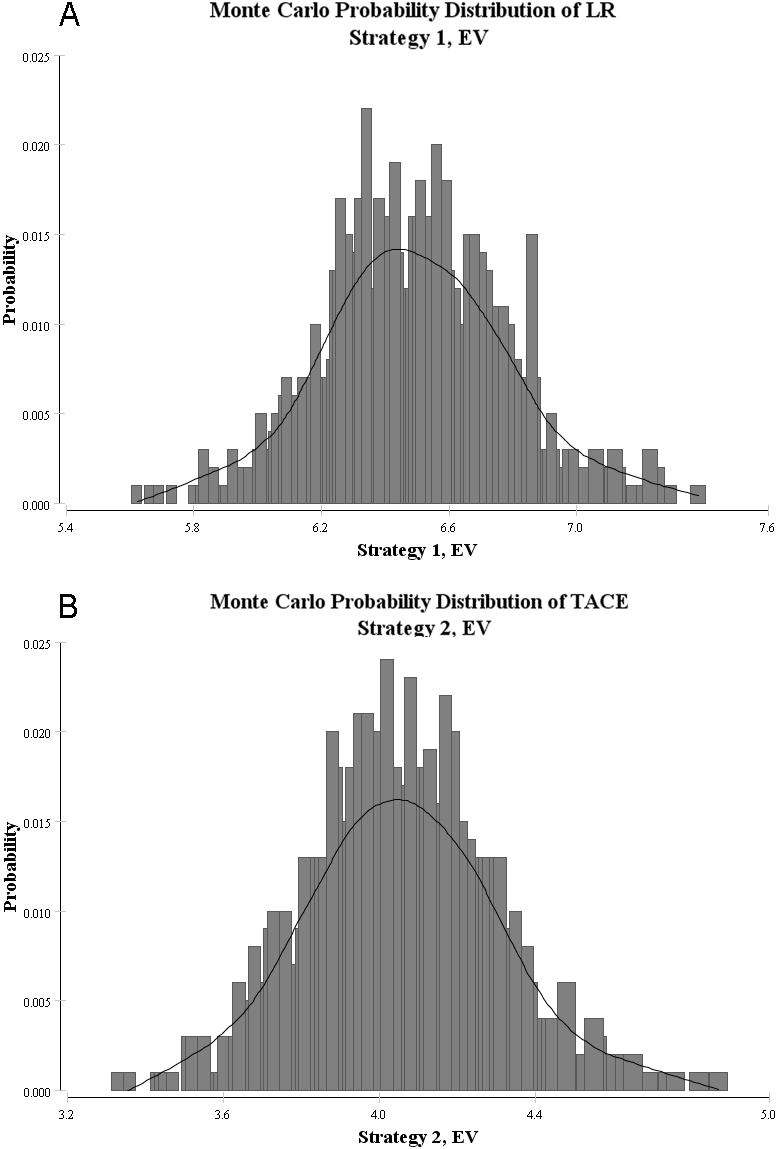

Supplement: Supplementary file 3 [file CAM4-8-1530-s003.tif]
