# Supplementary material for: Liver resection versus transarterial chemoembolization for the treatment of intermediate‐stage hepatocellular carcinoma
Source: Cancer Med. 2019 Mar 12;8(4):1530–9. doi: 10.1002/cam4.2038 (PMC6488138; doi:10.1002/cam4.2038)
Supplement: Supplementary file 4 [file CAM4-8-1530-s004.docx]

**Liver Resection versus Transarterial Chemoembolization for the Treatment of Intermediate-Stage Hepatocellular Carcinoma**

**Supplementary Methods**

**1. Literature Selection**

1.1. Study Strategy

Literatures were retrieved from two databases of PubMed and Cochrane Library with the latest search occurring on July 01, 2016. The following search terms were used, as either Medical Subject Headings (MeSH) or text words: hepatocellular carcinoma, liver cancer, primary liver carcinoma, liver cell carcinoma, hepatectomy, liver resection, liver surgery, hepatic resection, resection, surgery, surgical therapy, transarterial chemoembolization, TACE, transarterial embolization, TAE. Boolean operators (AND, OR) were used in succession to create combinations to complete the search. Reference lists from the included studies were hand-searched to identify further relevant literatures. If data subsets were published in more than one article, only the most recent article was included. Citations were limited to those published in English and the search was not restricted by publication year. Two investigators independently searched all the eligible studies and a third individual was consulted when the two evaluators’ opinions differed.

1.2. Study Selection and Data Extraction

Studies were considered eligible if they met the following criteria: (i) the entire population or subpopulation were adult patients with intermediate-stage hepatocellular carcinoma (HCC) who underwent only liver resection (LR) or transarterial chemoembolization (TACE) as the initial treatment; (ii) any of the parameter estimates used in our model was reported, those without the associated probabilities were excluded; (iii) randomized trials, quasi-randomized trials, prospective or retrospective cohort studies were included whereas reviews, letters, case reports, editorials or comments and meeting abstracts were excluded. Subsequently, we extracted all the transition probabilities from the corresponding included articles. Two investigators independently evaluated all eligible studies and extracted their data. Disagreements were resolved with the discussion with a third investigator.

**2. Parameter Estimation**

Double arcsine transformations were performed on the extracted transition probabilities before pooling them for variances stabilization because the inverse variance weight in the pooling is suboptimal when low prevalence rates are involved and the transformed probabilities are weighted very slightly towards 50%, making it feasible to include studies with prevalence rates of zero.^1^ The Wilson score method was also used to calculate the 95% confidence intervals (CIs) of these probabilities because of the values below zero produced by the asymptotic method.^2^ After obtaining the above estimates, STATA software (Stata Corp., College Station, TX, USA) was used to pool the data using the random-effect model. SAS 9.2 (SAS Institute Inc., Cary, NC, USA) was used to apply the Wilson score method and calculate the 95% CIs.

**3. Summary of Transition Probabilities and Assumptions**

For disease-free patients, the estimated annual mortality was derived from the sum of the annual mortality of general population and the liver-related annual mortality of cirrhotic patients.^3^ According to the average age of patients in the included studies, we assumed the mean age of this cohort to be 60-65 years with the annual age-related mortality of 0.055.^4^ The reported cirrhosis-related mortality was 0.027.^5^ In terms of the non-liver related and tumor-free liver related annual mortality, they were estimated as Cho YK et al. reported.^3^ Due to the similar median survival of advanced stage patients after LR and TACE,^6-11^ we assumed the same annual mortality for progressive patients in both groups.

During the last 3 decades, the perioperative mortality reduced with the development in anesthesia and surgery-related therapies. The procedure-related mortality for intermediate stage patients ranged between 0.0% and 5.4%.^12-17^ Considering the decreased invasiveness of TACE over LR and the assumption of best situation given to TACE, the perioperative mortality for TACE was assumed to be 0.0%.

Incomplete resection was an important factor predicting unfavorable prognosis for LR treatment. In our study, incomplete resection was defined as microscopic tumor invasion of the resection margins (R1).^18^ The patients with R1 resection were assumed to transit to progressive state. The incomplete resection rate was reported to range from 6% to 21.6%,^16,19,20^ which were pooled as 11.4%.

The tumor recurrence after LR was common in HCC patients. Forner et al. pointed out that the recurrence of HCC complicated 70.0% of surgical cases at 5 years.^21^ The annual probability of recurrence after LR was pooled as 24.5% with a range between 18.6% and 30.3%,^15,20,22-25^ The progression rate of recurrent tumors was reported as approximately 23.5%.^15,26^

The treatment response of TACE was stratified into four conditions: complete response (CR), partial response (PR), stable disease (SD) and progressive disease (PD). The pooled rates were 15.6% for CR, 49.4% for PR, 20.2% for SD and 14.8% for PD.^27-34^ There were very few articles investigating the tumor recurrence of CR patients and the transitions among PR, SD and PD stage in detail.^35,36^ Though a section of patients within the Milan criteria were also enrolled in one paper, we believed that the presented data might be rational enough to be used in our model under the assumption of the best scenario given for TACE. The probability of CR patients with recurrent HCC transiting to PD was pooled as 12.7%. We assumed the same probability for PR or SD patients moving to the PD stage, although it might underestimate the incidence of progressive disease in TACE group to some extent.

**4. External Validation**

4.1. Patients

From January 2008 to February 2014, 1,735 consecutive patients with intermediate-stage HCC either received LR (n=701) or TACE (n=1034) in the First Affiliated Hospital of Sun Yat-sen University. Written informed consent had been obtained from all patients before treatment. The study was approved by the Institutional Ethical Committee. According to the criteria of the European Association for Study of the Liver.^21^ HCC was diagnosed as the followed: two imaging techniques showing typical features of HCC, or an imaging technique with a serum alpha fetoprotein (AFP) level >400 ng/dL, or a cytologic/histologic confirmation of HCC.

The inclusion criteria were as follows: (1) age between 18 and 75 years; (2) resectable tumor which was evaluated by our surgery team, defining as the feasibility of removing all macroscopic lesions with adequate remaining liver function; (3) 2 to 3 lesions with at least 1 >3 cm in diameter; or more than 3 lesions of any diameter; (4) Child-Pugh A/B liver function ; (5) no previous treatment; (6) an Eastern Cooperative Oncology Group performance status of 0/1.

The exclusion criteria were (1) radiologic or pathological evidence of vascular invasion or extrahepatic metastases; (2) severe coagulopathy (prothrombin activity <40% or aplatelet count of<40,000/mm3); (3) evidence of hepatic decompensation including refractory ascites, esophageal or gastric variceal bleeding, or hepatic encephalopathy; (4) obstructive jaundice; (5) an American Society of Anesthesiologists score of 3 or more; (6) present or past history of any other concurrent malignancies; (7) complicated with other severe diseases such as chronic kidney disease, cardiovascular disease, auto-immunological disease, etc; (8) contraindications to carboplatin, epirubicin, mitomycin, or lipiodol; (9) no follow-up data.

4.2. Treatment Procedure

LR and TACE were performed respectively according to the protocols we reported previously.^37,38^ LR was performed by surgeons with 10 to 40 years of experience under general anesthesia. Intraoperative US was routinely used to assist in operative evaluation. Anatomic resection was carried out in the form of segmentectomy or/and subsegmentectomy as reported by Makuuchi et al.^39^ In segmentectomy, the hepatic parenchyma was transacted at the intersegmental plane as described by Couinaud. If the hepatic parenchymal transaction plane needed to go beyond the intersegmental plane to achieve the desired extent of resection margin, the small portal branches supplying the liver parenchyma up to the intended transaction plane were punctured under US guidance and injected with methylthioninium chloride, and then liver subsegmentectomy was performed either alone or in combination with segmentectomy along the plane of demarcation as delineated by the injected methylthioninium chloride. Nonanatomic resection with a negative resection margin was performed when anatomical resection may have caused an inadequate liver remnant. Pringle’s maneuver was used if necessary with a clamp/unclamp time of 10 min/5 min, respectively. The resected specimens were histopathologically examined. According to the guidelines of the International Union Against Cancer (UICC), R0 and R1 resections were defined by the absence (tumor-free margin 1 mm for all detected lesions) or presence (tumor-free margin of 0 mm) of microscopic tumor invasion of the resection margins.^18^

TACE was performed as the follows.^38^ After performing arteriography and portography to confirm the lesion size and location, a microcatheter (Renegade, Boston Scientific, Natick, Mass; Progreat, Terumo, Tokyo, Japan) was inserted into the feeding arteries as selectively as possible through the lobar, segmental, or subsegmental arteries, dependent on the tumor distribution and hepatic functional reserve. Hepatic artery infusion chemotherapy was performed using 300 mg carboplatin (Bristol–Myers Squibb, New York, NY). Subsequently, chemolipiodolization was performed mixed with 5 ml of lipiodol (Lipiodol Ultra–Fluide; André Guerbet Laboratories, Aulnay–sous–Bois, France). According to the number and size of the lesions, and liver and kidney function of the patient, the chemotherapeutic agents, including epirubicin (50–100 mg), pirarubicin (30–50 mg), hydroxycamptothecine (10–30 mg) and fluorouracil (500–1000 mg), were determined by the multidisciplinary team. If residual flow remained after infusion of these agents, additional lipiodol was injected. Embolization was performed with absorbable gelatin sponge particles (Gelfoam; Hangzhou Bi–Trumed Biotech Co., Ltd., Hangzhou, Zhejiang, China) 350–560 μm in diameter. The injection was slowed or discontinued if reflux occurred. Patients were monitored carefully, and analgesia (morphine or meperidine) was administered if necessary.

4.3. Follow-up

The follow-up protocol was conducted as our previous article described.^38^ Efficacy of LR and TACE were evaluated by using enhanced dynamic computed tomography (CT) at four weeks after treatment. Thereafter, the patients were followed up once every 3 months for the ﬁrst two years, once every 6 months from 2 to 5 years and then once every 12 months after 5 years. At each follow-up visit, CEUS and blood tests including liver function tests and AFP were carried out. Contrast-enhanced CT was performed once every 6 months in the first 5 years. Chest radiography was performed once every 6 months. MRI and bone scintigraphy were performed when clinically indicated. All images were reviewed by two experienced radiologists independently and disagreement was resolved by discussion with the third specialist.

Repeated TACE was performed once every 3 to 4 months until one of the following outcomes was reached: (1) complete devascularization of tumor; (2) technical impossibility to embolize the residual tumor, for example tumor only supplied by extrahepatic collateral arteries; (3) development of contraindications to TACE; or (4) complete removal of tumor by subsequent curative therapies. In the situation of (2) or (3), patients were recommended to receive sorafenib. If they refused, the best supportive treatment was given. The choice of treatment for re-recurrent HCC was determined by the characteristics of the recurrent tumor, patient request, and the consensus opinion of our multidisciplinary treatment team.

The severity of all adverse events was evaluated according to the National Cancer Institute Common Terminology Criteria for adverse events.^40^ TACE-related death was defined as death within 30 days from the initial therapy. This study was censored on December 31, 2015.

4.4. Statistical Analysis

Continuous variables were presented as means±SD and categorical variables as numbers and percentages. Differences between the two arms were compared by the t test for continuous variables and χ^2^ test for categorical variables. Survival curves were generated by the Kaplan–Meier method and compared by the log-rank test. Subgroup survival analyses were also performed according to tumor size. The prognostic relevance of potential survival predictors was analyzed by univariate and multivariate Cox proportional hazards regression models. All significant variables identified in univariate model were entered into the multivariate analysis. Statistical signiﬁcance was considered as a two-sided P value of less than 0.05. Since patients were not assigned randomly to receive LR or TACE, but rather based on their clinicopathological characteristics and request, it was very likely that our two patient groups would present significant baseline differences that might confound our analysis of outcomes. Propensity score matching was performed to adjust variables to control selection bias due to the non-randomization of patients allocated in two groups. All the baseline variables listed in Table 2 were entered in the full non parsimonious model. The propensity score-matched pairs were created by matching LR and TACE on the logit of the propensity score using calipers of width equal to 0.1 of the standard deviation of the logit of the propensity score^41^. For the matched cohorts, baseline characteristics were compared again by the Wilcoxon signed rank testor the McNemar test (or conditional logistic regression). Survival analysis was also repeated in the matched subgroup. The above statistical analysis was performed by the SPSS 20.0 (SPSS Inc., Chicago, IL, USA) and the R program (R Foundation for Statistical Computing, Vienna, Austria).

**Supplementary Results**

1. **One-way and Two-way Sensitivity Analyses**

One–way sensitivity analyses for all the variables showed that the curves of expected survival representing LR treatment were always above those representing TACE without crossing points (**Figure S1**). This suggested that initial LR always had the survival benefit over TACE. Similar results could be observed in two–way sensitivity analyses (**Table S4**). For any of the two variables, there was no point of intersection between these two therapies as shown in the figures, even when comparing the most sensitive factors in both groups and assuming the best case scenario for TACE. Tornado diagrams showed that the top three sensitive factors on survival in LR group were disease-free survival, perioperative mortality, and incomplete resection rate, while the top three factors in the TACE group were CR rate, progressive disease (PD) rate, and probability of partial response (PR) transited to PD (**Figure S2**). All the above factors were associated with the corresponding initial treatment. Thus, the results of Tornado diagram were consistent with those of the sensitivity analyses that showed the survival outcomes were more sensitive to variables related to initial treatment options.

1. **Perioperative and Operative Data in the Overall Validation Cohort and Propensity-score-matched Validation Cohort**

For the overall validation cohort, the incomplete resection rate was 8.4% (59/701) and the perioperative mortality rate was 0.4% (3/701) (**Table S5**) in the LR group. No reoperation was performed. In the TACE group, the mean number of procedural sessions was 3.4 (range, 1–6). No patient died during the hospital stay (**Table S6)**. The probabilities of each tumor response stage in validation cohort were estimated as 7.6% (79/1034), 22.2% (230/1034), 49.0% (507/1034), and 21.1% (218/1034) for CR, PR, stable disease (SD) and PD, respectively (**Table S6**).

For the propensity-score-matched validation cohort, the incomplete resection rate was 5.5% (34/623) and the perioperative mortality rate was 0.3% (2/701) (**Table S5**) in the LR group. No reoperation was performed which was the same as our model simulated. In the TACE group, the mean number of procedural sessions was 3.2 (range, 1–6). No patient died during the hospital stay which was in accordance with our assumption that the procedure related mortality was 0.0% (**Table S6)**. The probabilities of each tumor response stage in validation cohort were estimated as 8.2% (51/623), 22.6% (141/623), 49.1% (306/623), and 20.1% (125/623) for CR, PR, SD and PD respectively while in Markov model were 17.0%, 52.0%, 20.2% and 14.8%, respectively (**Table S6**).

**References**

1. Freeman MF TJ. Transformations related to the angular and the square root. Ann Math Stats 1950:607-611.

2. Newcombe RG. Two-sided confidence intervals for the single proportion: comparison of seven methods. Stat Med 1998;17:857-872.

3. Cho YK, Kim JK, Kim WT, Chung JW. Hepatic resection versus radiofrequency ablation for very early stage hepatocellular carcinoma: a Markov model analysis. Hepatology 2010;51:1284-1290.

4. Department Of Health And Human Services NCFH, Statistics. National Vital Statistics Reports 2006;57(14). www.dhhs.gov.

5. Bruno S, Zuin M, Crosignani A, Rossi S, Zadra F, Roffi L, et al. Predicting mortality risk in patients with compensated HCV-induced cirrhosis: a long-term prospective study. Am J Gastroenterol 2009;104:1147-1158.

6. Fu SR, Zhang YQ, Li Y, Hu BS, He X, Huang JW, et al. Sorafenib continuation after first disease progression could reduce disease flares and provide survival benefits in patients with hepatocellular carcinoma: a pilot retrospective study. Asian Pac J Cancer Prev 2014;15:3151-3156.

7. Lee IC, Chen YT, Chao Y, Huo TI, Li CP, Su CW, et al. Determinants of survival after sorafenib failure in patients with BCLC-C hepatocellular carcinoma in real-world practice. Medicine (Baltimore) 2015;94:e688.

8. Llovet JM, Bustamante J, Castells A, Vilana R, Ayuso MC, Sala M, et al. Natural history of untreated nonsurgical hepatocellular carcinoma: rationale for the design and evaluation of therapeutic trials. Hepatology 1999;29:62-67.

9. Pawlik TM, Poon RT, Abdalla EK, Ikai I, Nagorney DM, Belghiti J, et al. Hepatectomy for hepatocellular carcinoma with major portal or hepatic vein invasion: results of a multicenter study. Surgery 2005;137:403-410.

10. Pinter M, Hucke F, Graziadei I, Vogel W, Maieron A, Konigsberg R, et al. Advanced-stage hepatocellular carcinoma: transarterial chemoembolization versus sorafenib. Radiology 2012;263:590-599.

11. Wada Y, Takami Y, Tateishi M, Ryu T, Mikagi K, Saitsu H. The Efficacy of Continued Sorafenib Treatment after Radiologic Confirmation of Progressive Disease in Patients with Advanced Hepatocellular Carcinoma. PLoS One 2016;11:e146456.

12. Jianyong L, Lunan Y, Wentao W, Yong Z, Bo L, Tianfu W, et al. Barcelona clinic liver cancer stage B hepatocellular carcinoma: transarterial chemoembolization or hepatic resection? Medicine (Baltimore) 2014;93:e180.

13. Lin CT, Hsu KF, Chen TW, Yu JC, Chan DC, Yu CY, et al. Comparing hepatic resection and transarterial chemoembolization for Barcelona Clinic Liver Cancer (BCLC) stage B hepatocellular carcinoma: change for treatment of choice? World J Surg 2010;34:2155-2161.

14. Torzilli G, Donadon M, Marconi M, Palmisano A, Del FD, Spinelli A, et al. Hepatectomy for stage B and stage C hepatocellular carcinoma in the Barcelona Clinic Liver Cancer classification: results of a prospective analysis. Arch Surg 2008;143:1082-1090.

15. Wang BW, Mok KT, Liu SI, Chou NH, Tsai CC, Chen IS, et al. Is hepatectomy beneficial in the treatment of multinodular hepatocellular carcinoma? J Formos Med Assoc 2008;107:616-626.

16. Yin L, Li H, Li AJ, Lau WY, Pan ZY, Lai EC, et al. Partial hepatectomy vs. transcatheter arterial chemoembolization for resectable multiple hepatocellular carcinoma beyond Milan Criteria: a RCT. J Hepatol 2014;61:82-88.

17. Zhong JH, Xiang BD, Gong WF, Ke Y, Mo QG, Ma L, et al. Comparison of long-term survival of patients with BCLC stage B hepatocellular carcinoma after liver resection or transarterial chemoembolization. PLoS One 2013;8:e68193.

18. Sobin LH WC. TNM Classification of Malignant Tumours. 6th ed. Hoboken, NJ: John Wiley &#38; Sons; 2002.

19. Lim C, Mise Y, Sakamoto Y, Yamamoto S, Shindoh J, Ishizawa T, et al. Above 5 cm, size does not matter anymore in patients with hepatocellular carcinoma. World J Surg 2014;38:2910-2918.

20. Ng KK, Vauthey JN, Pawlik TM, Lauwers GY, Regimbeau JM, Belghiti J, et al. Is hepatic resection for large or multinodular hepatocellular carcinoma justified? Results from a multi-institutional database. Ann Surg Oncol 2005;12:364-373.

21. Bruix J, Sherman M, Llovet JM, Beaugrand M, Lencioni R, Burroughs AK, et al. Clinical management of hepatocellular carcinoma. Conclusions of the Barcelona-2000 EASL conference. European Association for the Study of the Liver. J Hepatol 2001;35:421-430.

22. Chang WT, Kao WY, Chau GY, Su CW, Lei HJ, Wu JC, et al. Hepatic resection can provide long-term survival of patients with non-early-stage hepatocellular carcinoma: extending the indication for resection? Surgery 2012;152:809-820.

23. Ciria R, Lopez-Cillero P, Gallardo AB, Cabrera J, Pleguezuelo M, Ayllon MD, et al. Optimizing the management of patients with BCLC stage-B hepatocellular carcinoma: Modern surgical resection as a feasible alternative to transarterial chemoemolization. Eur J Surg Oncol 2015;41:1153-1161.

24. Qian H, Wei M, Qiu H, Wu J, Liu B, Lyu A, et al. A scoring system for prediction of early recurrence after liver resection for Barcelona Clinic Liver Cancer stage B hepatocellular carcinoma. Chin Med J (Engl) 2014;127:4171-4176.

25. Wang X, Wang Z, Wu L. Combined measurements of tumor number and size helps estimate the outcome of resection of Barcelona clinic liver cancer stage B hepatocellular carcinoma. BMC Surg 2016;16:22.

26. Luo J, Peng ZW, Guo RP, Zhang YQ, Li JQ, Chen MS, et al. Hepatic resection versus transarterial lipiodol chemoembolization as the initial treatment for large, multiple, and resectable hepatocellular carcinomas: a prospective nonrandomized analysis. Radiology 2011;259:286-295.

27. El FA, Ertle J, El DA, Shaker MK, Dechene A, Abdella H, et al. In intermediate stage hepatocellular carcinoma: radioembolization with yttrium 90 or chemoembolization? Liver Int 2015;35:627-635.

28. Heng-jun G, Yao-jun Z, Min-shan C, Mei-xian C, Jun-ting H, Li X, et al. Rationality and effectiveness of transarterial chemoembolization as an initial treatment for BCLC B stage HBV-related hepatocellular carcinoma. Liver Int 2014;34:612-620.

29. Llovet JM, Real MI, Montana X, Planas R, Coll S, Aponte J, et al. Arterial embolisation or chemoembolisation versus symptomatic treatment in patients with unresectable hepatocellular carcinoma: a randomised controlled trial. Lancet 2002;359:1734-1739.

30. Lo CM, Ngan H, Tso WK, Liu CL, Lam CM, Poon RT, et al. Randomized controlled trial of transarterial lipiodol chemoembolization for unresectable hepatocellular carcinoma. Hepatology 2002;35:1164-1171.

31. Nishikawa H, Osaki Y, Kita R, Kimura T, Ohara Y, Takeda H, et al. Comparison of transcatheter arterial chemoembolization and transcatheter arterial chemotherapy infusion for patients with intermediate-stage hepatocellular carcinoma. Oncol Rep 2014;31:65-72.

32. Shim JH, Lee HC, Kim SO, Shin YM, Kim KM, Lim YS, et al. Which response criteria best help predict survival of patients with hepatocellular carcinoma following chemoembolization? A validation study of old and new models. Radiology 2012;262:708-718.

33. Song MJ, Chun HJ, Song DS, Kim HY, Yoo SH, Park CH, et al. Comparative study between doxorubicin-eluting beads and conventional transarterial chemoembolization for treatment of hepatocellular carcinoma. J Hepatol 2012;57:1244-1250.

34. Wu L, Yang YF, Ge NJ, Shen SQ, Liang J, Wang Y, et al. Hepatic artery injection of (1)(3)(1)I-labelled metuximab combined with chemoembolization for intermediate hepatocellular carcinoma: a prospective nonrandomized study. Eur J Nucl Med Mol Imaging 2012;39:1306-1315.

35. Terzi E, Golfieri R, Piscaglia F, Galassi M, Dazzi A, Leoni S, et al. Response rate and clinical outcome of HCC after first and repeated cTACE performed "on demand". J Hepatol 2012;57:1258-1267.

36. Xu L, Peng ZW, Chen MS, Shi M, Zhang YJ, Guo RP, et al. Prognostic nomogram for patients with unresectable hepatocellular carcinoma after transcatheter arterial chemoembolization. J Hepatol 2015;63:122-130.

37. Peng ZW, Lin XJ, Zhang YJ, Liang HH, Guo RP, Shi M, et al. Radiofrequency ablation versus hepatic resection for the treatment of hepatocellular carcinomas 2 cm or smaller: a retrospective comparative study. Radiology 2012;262:1022-1033.

38. Peng ZW, Zhang YJ, Chen MS, Xu L, Liang HH, Lin XJ, et al. Radiofrequency ablation with or without transcatheter arterial chemoembolization in the treatment of hepatocellular carcinoma: a prospective randomized trial. J Clin Oncol 2013;31:426-432.

39. Makuuchi M, Hasegawa H, Yamazaki S. Ultrasonically guided subsegmentectomy. Surg Gynecol Obstet 1985;161:346-350.

40. Institute NC. Common Toxicity Criteria, Version 4.0: Cancer Therapy Evaluation Program, 2009. http://evs.nci.nih.gov/ftp1/CTCAE/CTCAE_4.03_2010-06-14_QuickReference_5x7.pdf.

**Table S1.** Detailed Characteristics of Studies on LR Treatment Included in the Model

| Author Year | Sample  size | Age  (years) | Child-Pugh  Class A (%) | Perioperative mortality | Incomplete resection rate | 5-year recurrence rate | 5-year disease free survival | Progression rate of recurrent HCC |
| --- | --- | --- | --- | --- | --- | --- | --- | --- |
| Torzilli G^7^, 2008 | 24 | 66 | - | 0 | - | - | - | - |
| Wang BW^36^, 2008 | 112 | 59 | - | 0.027 | - | - | 0.184 | 0.243 |
| Lin CT^19^, 2010 | 93 | 59 | 100% | 0.054 | - | - | - | - |
| Luo J^33^, 2011 | 85 | 47.5 | 70.6% | - | - | - | - | 0.224 |
| Chang WT^29^, 2012 | 318 | 60.4 | 97.8% | - | - | 0.681 | - | - |
| Lim C^32^, 2014 | 41 | 62 | 75.6% | - | 0.100 | - | - | - |
| Yin L^22^, 2014 | 88 | 51.6 | 98.9% | 0.011 | 0.216 | - | - |  |
| Ciria R^30^, 2015 | 36 | 67 | 88.9% | - | - | 0.444 | - | - |
| Qian H^35^,2014 | 104 | 52 | - | - | - | - | 0.375 | - |
| Wang X^37^,2016 | 78 | 56 | 97.4% | - | - | - | 0.166 | - |
| Ng KK^34^,2005 | 380 | 58.4 | 95% | - | 0.06 | - | 0.26 | - |
| Jianyong L^31^,2014 | 433 | 53 | 75.8% | 0.023 | - | - | - | - |
| Zhong JH^21^,2013 | 257 | 46.8 | - | 0.031 | - | - | - | - |

Abbreviations: LR, liver resection; HCC, hepatocellular carcinoma.

**Table S2.** Detailed characteristics of studies on TACE treatment included in the model

| Author Year | Sample  size | Age  (years) | Child-Pugh  Class A (%) | CR rate after TACE | SD rate after TACE |  | PD rate after TACE | Recurrence rate after CR | Transition rate from CR to PD | Transition rate from PR to PD | Transition rate from SD to PD |
| --- | --- | --- | --- | --- | --- | --- | --- | --- | --- | --- | --- |
| Llovet JM^12^, 2002 | 40 | 63 | 77.5% | 0.025 | 0.425 |  | 0.225 | - | - | - | - |
| Lo CM^40^, 2002 | 40 | 62 | - | 0 | 0.175 |  | 0.100 | - | - | - | - |
| Song MJ^43^, 2012 | 41 | 59.4 | - | 0.073 | 0.390 |  | 0.269 | - | - | - | - |
| Wu L^45^, 2012 | 70 | 46 | 81.4% | 0.243 | 0.157 |  | 0.157 | - | - | - | - |
| Shim JH^42^, 2012 | 332 | 62 | 100.0% | 0.406 | 0.072 |  | 0.090 | - | - | - | - |
| Terzi E^44^, 2012 | 151 | 65 | 64.2% | - | - |  | - | 0.610 | 0.100 | 0.100 | 0.100 |
| Nishikawa H^41^, 2013 | 145 | 72.5 | 69.0% | 0.207 | 0.193 |  | 0.007 | - | - | - | - |
| Heng-jun G^39^, 2014 | 1516 | 60 | 91.7% | 0.216 | 0.263 |  | 0.132 | - | - | - | - |
| El FA^38^, 2015 | 42 | 58.3 | 78.6% | 0.050 | 0.210 |  | 0.290 | - | - | - | - |
| Xu L^46^,2015 | 1740 | 57 | 96% | - | - |  | - | 0.70 | 0.14 | 0.14 | 0.14 |

Abbreviations: TACE, transarterial chemoembolization; CR, complete remission; SD, stable disease; PD, progressive disease; PR, partial remission.

**Table S3.** Detailed Characteristics of Studies on Common Probabilities Included in the Model

| Author Year | Sample  size | Age  (years) | 3-year survival rate | Median survival of progressive HCC (months) | Mortality of cirrhotic patients | Age-specific Mortality |
| --- | --- | --- | --- | --- | --- | --- |
| Llovet JM^51^,1999 | 54 | - | 8.0% | - | - | - |
| Pawlik TM^52^,2005 | 102 | 59 | - | 11.0 | - | - |
| Bruno S^47^,2009 | 352 | - | - | - | 0.027 | - |
| Heron M^48^, 2009 | - | - | - | - | - | 0.055 |
| Pinter M^53^,2012 | 97 | 60 | - | 9.2 | - | - |
| Wada Y^54^,2016 | 39 | 69.7 | - | 4.5 | - | - |
| Lee IC^50^,2015 | 149 | 61.9 | - | 4.6 | - | - |
| Fu SR^49^,2014 | 24 | 57 | - | 8.6 | - | -- |

Abbreviations: HCC, hepatocellular carcinoma

**Table S4.** The text report of two-way sensitivity analysis for the most sensitive variables in LR group and TACE group

| TDiseaseCR/LDFSDie | | 0.057 | 0.064 | 0.071 | 0.078 | 0.085 | 0.092 | 0.099 | 0.106 | 0.114 |
| --- | --- | --- | --- | --- | --- | --- | --- | --- | --- | --- |
| 0.025 | LR | 7.221 | 6.970 | 6.734 | 6.511 | 6.300 | 6.100 | 5.912 | 5.734 | 5.566 |
|  | TACE | 3.359 | 3.359 | 3.359 | 3.359 | 3.359 | 3.359 | 3.359 | 3.359 | 3.359 |
| 0.073 | LR | 7.256 | 7.005 | 6.769 | 6.546 | 6.335 | 6.135 | 5.947 | 5.769 | 5.601 |
|  | TACE | 3.614 | 3.614 | 3.614 | 3.614 | 3.614 | 3.614 | 3.614 | 3.614 | 3.614 |
| 0.120 | LR | 7.289 | 7.039 | 6.802 | 6.579 | 6.368 | 6.169 | 5.980 | 5.802 | 5.634 |
|  | TACE | 3.856 | 3.856 | 3.856 | 3.856 | 3.856 | 3.856 | 3.856 | 3.856 | 3.856 |
| 0.168 | LR | 7.321 | 7.070 | 6.834 | 6.611 | 6.400 | 6.200 | 6.012 | 5.834 | 5.666 |
|  | TACE | 4.087 | 4.087 | 4.087 | 4.087 | 4.087 | 4.087 | 4.087 | 4.087 | 4.087 |
| 0.216 | LR | 7.351 | 7.101 | 6.864 | 6.641 | 6.430 | 6.231 | 6.042 | 5.864 | 5.696 |
|  | TACE | 4.307 | 4.307 | 4.307 | 4.307 | 4.307 | 4.307 | 4.307 | 4.307 | 4.307 |
| 0.263 | LR | 7.380 | 7.130 | 6.893 | 6.670 | 6.459 | 6.260 | 6.071 | 5.893 | 5.725 |
|  | TACE | 4.517 | 4.517 | 4.517 | 4.517 | 4.517 | 4.517 | 4.517 | 4.517 | 4.517 |
| 0.311 | LR | 7.408 | 7.157 | 6.921 | 6.698 | 6.487 | 6.287 | 6.099 | 5.921 | 5.753 |
|  | TACE | 4.717 | 4.717 | 4.717 | 4.717 | 4.717 | 4.717 | 4.717 | 4.717 | 4.717 |
| 0.358 | LR | 7.434 | 7.184 | 6.947 | 6.724 | 6.513 | 6.314 | 6.126 | 5.948 | 5.779 |
|  | TACE | 4.909 | 4.909 | 4.909 | 4.909 | 4.909 | 4.909 | 4.909 | 4.909 | 4.909 |
| 0.406 | LR | 7.460 | 7.209 | 6.973 | 6.750 | 6.539 | 6.339 | 6.151 | 5.973 | 5.805 |
|  | TACE | 5.092 | 5.092 | 5.092 | 5.092 | 5.092 | 5.092 | 5.092 | 5.092 | 5.092 |

Abbreviations: LR, liver resection; TACE, transarterial chemoembolization.

Note the expected overall survival in HR group and TACE group when it referred to the highest mortality for HR group and the highest CR rate for TACE group.

**Table S5.** Operative Details and perioperative outcomes

|  | Before matching(n=701) | After matching(n=623) |
| --- | --- | --- |
| Type of hepatectomy |  |  |
| 1 segment | 46 (6.6%) | 35 (5.6%)) |
| 2 segment | 318 (45.3%) | 306 (49.1%)) |
| 3 segment | 207 (29.5%) | 189 (30.3%)) |
| Left lobe | 48 (6.8%) | 41(6.6%)) |
| Right lobe | 64 (9.1%)) | 52 (8.3%)) |
| R0/R1 resection | 642/59 | 589/34 |
| Operating time (min) | 141.5±32.2 | 140.2±34.1 |
| Intraoperative blood loss (ml) | 306.5(120-7200) | 320.1(120-7200) |
| Blood transfusion (n) | 101(14.4%)) | 89 (14.3%)) |
| Time of Pringle maneuver (min) | 17.5±5.0 | 16.1±5.5 |
| Gastrointestinal hemorrhage | 15(2.1%)) | 10 (1.6%)) |
| Bile leakage | 29 (4.1%)) | 19 (3.0%)) |
| Pulmonary complications | 20 (2.9%)) | 15 (2.4%)) |
| Spontaneous bacterial peritonitis | 7 (1.0%)) | 4 (0.6%)) |
| Perioperative death | 3(0.4%)) | 2 (0.3%)) |
| Liver failure | 10 (1.4%)) | 7 (1.1%)) |

Abbreviations: LR, liver resection.

**Table S6.** Post-treatment Outcomes of TACE

|  | Before matching(n=1034) | After matching(n=623) |
| --- | --- | --- |
| Mean sessions | 3.4 | 3.2 |
| Hospital mortality | 0 (0.0%) | 0 (0.0%) |
| Liver failure | 4 (0.4%) | 2 (0.3%) |
| Increase in ALT/AST | 605 (58.5%) | 378 (60.7%) |
| Cholecystitis | 3 (0.3%) | 1(0.2%) |
| Fever  (temperature >38.5°C) | 51(4.9%) | 38 (6.1%) |
| Pain | 356 (34.4%) | 201 (32.3%) |
| Decrease in albumin | 278 (26.9%) | 176 (28.3%) |
| Increase in bilirubin | 246 (23.8%) | 141(22.6%) |
| Leukopenia | 389 (37.6%) | 212 (34.0%) |
| Nausea/vomiting | 896 (86.7%) | 478 (76.7%) |
| Tumor response |  |  |
| CR | 79 (7.6%) | 51(81.9%) |
| PR | 230 (22.2%) | 141(22.6%) |
| SD | 507 (49.0%) | 306 (49.1%) |
| PD | 218 (21.1%) | 125 (20.1%) |
| Abbreviations: TACE, transarterial chemoembolization; ALT, alanine aminotransferase; AST, aspartate aminotransferase; CR, complete remission; PR, partial remission; SD, stable disease; PD, progressive disease. | | |

**Table S7.** Univariate and Multivariate Analysis of Overall Survival for All Patients

|  | Univariate analysis | Multivariate analysis | | |
| --- | --- | --- | --- | --- |
| Variables | *P* value | *P* value | HR | 95%CI |
| Age (y), ≤60 or >60 | 0.234 |  |  |  |
| Sex(M/F) | 0.201 |  |  |  |
| Hepatitis B (+/-) | 0.354 |  |  |  |
| Hepatitis C (+/-) | 0.278 |  |  |  |
| ECOG (0/1) | 0.699 |  |  |  |
| Portal hypertension (yes/no) | 0.500 |  |  |  |
| Cirrhosis(yes/no) | 0.743 |  |  |  |
| Child Pugh classification (A/B) | 0.225 |  |  |  |
| ICGR15(%), ≤10 or >10 | 0.301 |  |  |  |
| Tumor size, ≤5 or >5cm | 0.003 |  |  |  |
| Tumor number, ≤2 or >2 | 0.001 | 0.002 | 1.238 | 1.095-1.400 |
| AFP (ng/ml) , ≤400 or >400 | <0.001 | <0.001 | 1.485 | 1.237-1.783 |
| GGT, (u/L), ≤50 or >50 | 0.987 |  |  |  |
| ALT (u/L), ≤40 or >40 | 0.923 |  |  |  |
| Albumin (g/L), ≤35 or >35 | 0.423 |  |  |  |
| TBIL (umol/L), ≤20 or >20 | 0.562 |  |  |  |
| Platelet count (10^9^/L), ≤100 or >100 | 0.100 |  |  |  |
| Hemoglobin (g/L), ≤110 or >110 | 0.221 |  |  |  |
| White blood cell (10^9^/L) ≤4 or >4 | 0.865 |  |  |  |
| Distribution of tumor | 0.675 |  |  |  |
| Treatment allocation, TACE vs.LR | <0.001 | <0.001 | 1.497 | 1.328-1.688 |
| Abbreviations: HR, hazard ratio; CI, confidence interval; GGT, γ-glutamyltranspeptidase; ALT, alanine aminotransferase; TBIL, total bilirubin; ICGR15, indocyanine green retention rate in 15 minutes; AFP, alpha-fetoprotein; TACE, transarterial chemoembolization; LR, liver resection; ECOG, Eastern Cooperative Oncology Group. | | | | |
